# Polyacrylate–Peptide Antigen Conjugate as a Single-Dose Oral Vaccine against Group A *Streptococcus*

**DOI:** 10.3390/vaccines8010023

**Published:** 2020-01-13

**Authors:** Mohammad Omer Faruck, Lili Zhao, Waleed M. Hussein, Zeinab G. Khalil, Robert J. Capon, Mariusz Skwarczynski, Istvan Toth

**Affiliations:** 1 School of Chemistry and Molecular Biosciences, The University of Queensland, St Lucia, Brisbane, QLD 4072, Australia; m.faruck@uq.edu.au (M.O.F.); lili.zhao@uq.edu.au (L.Z.); w.hussein@uq.edu.au (W.M.H.); 2 Pharmaceutical Organic Chemistry Department, Faculty of Pharmacy, Helwan University, Helwan 11795, Egypt; 3 Institute for Molecular Bioscience, The University of Queensland, St. Lucia, QLD 4072, Australia; z.khalil@imb.uq.edu.au (Z.G.K.); r.capon@imb.uq.edu.au (R.J.C.); 4 School of Pharmacy, The University of Queensland, Woolloongabba, Brisbane, QLD 4102, Australia

**Keywords:** peptide vaccine, poly (methyl acrylate), oral delivery, nanoparticles, polymer–peptide conjugate, Group A *Streptococcus*

## Abstract

Group A *Streptococcus* (GAS)-associated rheumatic heart disease is a leading cause of death caused by GAS infection. While antibiotics can treat the infection in most cases, growing antibiotic resistance, late medical intervention, and recurrent infection are major obstacles to the effective treatment of GAS-associated diseases. As GAS infection typically originates from the bacterial colonization of mucosal tissue in the throat, an oral vaccine that can generate both systemic and mucosal immune responses would solve problems associated with traditional medical interventions. Moreover, orally delivered vaccines are more easily administered and less expensive for mass immunization. In this study, the B-cell epitope J8, derived from GAS M protein, and universal T-helper Pan HLA-DR-binding epitope peptide (PADRE), were conjugated to poly (methyl acrylate) (PMA) to form a self-assembled nanoparticle vaccine candidate (PMA-P-J8). Strong systemic and mucosal immune responses were induced upon single oral immunization of mice with the conjugate. The antibodies generated were opsonic against GAS clinical isolates as measured after boost immunization. Thus, we developed a simple conjugate as an effective, adjuvant-free oral peptide-based vaccine.

## 1. Introduction

Vaccination is the most successful and cost-effective public health intervention to counter infectious diseases and related mortality. Conventional vaccines consisting of killed or attenuated live pathogens are effective; however, undesired side-effects such as autoimmune and allergic responses and inflammation limit the use of whole organisms in modern vaccines [1,2]. To overcome these issues, most vaccine research has shifted to the development of subunit-based vaccines that include limited microbial components. Subunit vaccines are composed usually of protein or peptide antigens derived from pathogens [3,4]. The use of only selected antigens and the elimination of redundant components improves a vaccine’s safety profile; however, it also greatly reduces immunogenicity. Therefore, adjuvants (immune stimulants) are generally required for subunit vaccines [5,6].

Parenteral (intramuscular or subcutaneous) immunization is considered to be the most effective vaccine administration strategy, but it also results in low patient compliance, carries a risk of needle-associated infections, and requires skilled medical personnel for administration, among other limitations. In contrast, oral delivery is the most desirable route, especially for mass immunizations. Importantly, mucosal vaccine delivery, including oral strategies, not only generates systemic immune responses but can also trigger mucosal immunity, which prevents the initial mucosal colonization of a pathogen [7,8,9]. The challenge with oral administration is that vaccine components such as proteins, polysaccharides, and peptides are extremely labile and are degraded while passing through the stomach and gut [10]. Antigen dilution is also an important problem for oral vaccine delivery due to the large surface area of the gastrointestinal tract, resulting in the need for higher active ingredient quantities and multiple doses. Multiple dosing is not only inconvenient and more expensive, but it can also trigger oral tolerance to the delivered antigen. Therefore, the development of appropriate delivery systems that can protect antigens from degradation and help trigger systemic and mucosal immune responses is in high demand. 

*Streptococcus pyogenes* is a Gram-positive coccus species that colonizes the pharynx and skin; it is often referred to as Group A *Streptococcus* (GAS) [11]. GAS is responsible for a wide range of human diseases, including uncomplicated pharyngitis, impetigo, pyoderma, necrotizing fasciitis, cellulitis, septic arthritis, osteomyelitis, bacteremia [12,13], and post-infection complications, including acute rheumatic fever (ARF), rheumatic heart disease (RHD), and poststreptococcal glomerulonephritis [14]. RHD alone is responsible for 0.3 to 1.4 million death per year [15,16]. Current treatment for RHD includes antibiotic therapy with penicillin, erythromycin, or cephalosporin [17]. However, the development of allergic reactions to penicillin and the emergence of bacterial resistance to erythromycin limits the scope of antibiotic therapy [18]. The risk of a resurgence of invasive diseases and poor disease management in developing countries also dictates the need for better solutions to control GAS infection. Unfortunately, no commercial vaccine is available for GAS infection [19,20]. 

The virulence of GAS is determined by a variety of the pathogen’s components, including Group A streptococcal carbohydrate, streptococcal fibronectin-binding proteins, cysteine protease, C5a peptidase, Sfb1, and surface M protein [21]. Surface M protein is considered to be a particularly important virulence determinant in GAS infection, and has become a leading target in vaccine development strategies. The M protein has a coiled-coil configuration, and mainly consists of three domains: a highly variable repeat/N-terminal domain, a B-repeat central domain, and a conserved C/D-repeat domain [22]. The direct use of M protein in vaccine development was rejected due to the potential for cross-reactivity with heart muscle [23]. However, advances in epitope mapping have enabled the identification of several B-cell epitopes based on M protein [24]. New-generation GAS vaccine designs are focusing on the conserved C-repeat region epitopes, as they have shown potential for providing protection against most GAS strains without inducing autoimmune responses [20,25,26,27]. The α-helical B-cell epitope J8 (QAEDKVKQSREAKKQVEKALKQLEDKVQ) derived from M protein has recently passed Phase I clinical trials [28,29]. Early attempts to develop orally administered vaccines based on M-protein-conserved B-cell epitopes were only partially successful. Oral administration of lipidated antigens resulted in moderate humoral immune responses only, even with six or seven boosts and the use of alkalizers [30,31]. While a lipidated antigen incorporated into liposomes coated by alginate and mucoadhesive chitosan triggered a relatively strong immune response, the required dose and number of immunizations was still high (100 μg × 4) [32].

**Figure 1:**
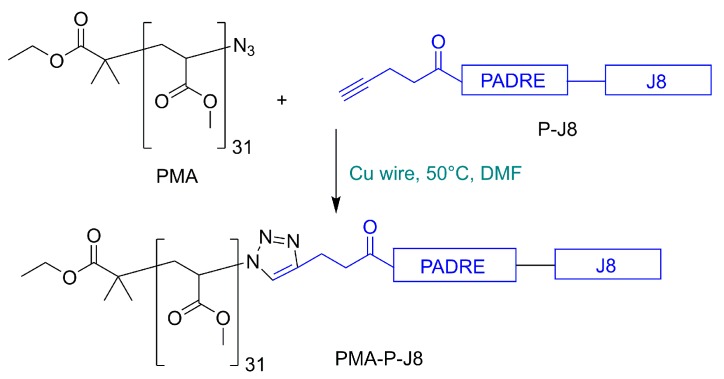
Schematic illustration of the synthesis of the vaccine candidate containing J8 B-cell epitope, PADRE universal T-helper (AKFVAAWTLKAAA) epitope, and poly (methyl acrylate) (PMA), PMA-P-J8.

In this study, we synthesized a conjugate containing J8 B-cell epitope, PADRE universal T-helper (AKFVAAWTLKAAA) epitope, and poly (methyl acrylate) (PMA) (Figure 1), which self-assembled into nanoparticles. While linear and branched polyacrylates have been used widely in vaccine delivery to generate systemic cellular and humoral immune responses [33,34,35,36,37,38,39,40], this is the first report of the use of polyacrylate for oral vaccine delivery. The developed peptide–polymer conjugate induced the production of systemic and mucosal antibodies, even after single oral immunization. 

## 2. Materials and Methods 

### 2.1. Materials 

All chemicals used in this study were analytical grade. Protected L-amino acids were purchased from Novabiochem (Laufelfingen, Switzerland). Rink amide MBHA resin, *N*,*N*-diisopropylethylamine (DIPEA), dicholoromethane (DCM), piperidine, and trifluoroacetic acid (TFA) were purchased from Merck (Hohbrunn, Germany). 1-[Bis(dimethylamino)methylene]-1*H*-1,2,3-triazolo [4,5-b]pyridinium-3-oxid hexafluorophosphate (HATU) was purchased from Mimotopes (Melbourne, Australia). Pentanoic acid, poly (methyl acrylate)-azide terminal (PMA), secondary antibody IgG, and IgA were purchased from Sigma Aldrich (Castle Hill, NSW, Australia).

### 2.2. Equipment

Electrospray Ionisation Mass Spectrometry (ESI-MS) analysis was done using a Perkin Elmer Sciex API3000 Instrument (Applied Biosystem/MDS Sciex, Toronto, ON, Canada) with Analytes 1.4 software. Analytical RP-HPLC analysis was done on a Shimadzu (Kyoto, Japan) instrument (DGU-20A5, LC-20AB, SIL-20ACHT, SPD-M10AVP) with a flow rate of 1 mL/min and detection at 214 nm. Analytical HPLC analysis was done with a 0%–100% gradient of analytical grade solvent A (0.1% TFA in water) to solvent B (90% MeCN, 10% water, 0.1% TFA) over 50 min and a Vydac analytical C18 column (218TP54; 5 µm, 4.6 mm × 250 mm). Preparative RP-HPLC analysis was done with a Shimadzu Instrument (Tokyo, Japan) at a flow rate of 20 mL/min and a Vydac C18 column, with detection at 230 nm. Particle size was measured by dynamic light scattering (DLS; Malvern Zetasizer Nano Series with DTS software). Transmission electron microscopy (TEM; HT7700 Exalens, HITACHI Ltd., Tokyo, Japan) was performed at the Australian Microscopy & Microanalysis Research Facility, Centre for Microscopy and Microanalysis, The University of Queensland (UQ). Element microanalysis (EA) was performed in the School of Chemistry and Molecular Biosciences, UQ, using a FLASH 2000 instrument (Thermo Fisher Scientific, Waltham, MA, USA). 

### 2.3. Synthesis of 4-Pentynoyl Derivative of PADRE-J8 Peptide

PADRE-J8 was synthesized by 9-fluorenylmethyloxycarbonyl (Fmoc) solid-phase peptide synthesis (SPPS) on a 0.2 mmol scale. Resin was pre-swelled in DMF overnight and deprotection of Fmoc was done twice with 20% piperidine in DMF first for 10 min, then for 15 min. Amino acids (4.2 equivalent) were activated by HATU in DMF (4.0 equivalent) and DIPEA (5.2 equivalent). Each amino acid was coupled twice, first for 10 min, then for 30 min. This process was repeated until the desired sequence was complete. Subsequently, 4-pentynoic acid was coupled on a 0.2 mmol scale to add an alkyne moiety at the *N*-terminus of the peptide. The resin was washed with DMF, DCM, and methanol, then dried in the desiccator overnight. The peptide was cleaved from the resin using a mixture of trifluoroacetic acid (TFA):triisopropylsilane (TIPS):water (95:2.5:2.5). The TFA was then removed by evaporation under reduced pressure. The peptide was precipitated in cold diethyl ether and dissolved in a mixture of acetonitrile and water (50:50) containing 0.1% TFA. Crude 4-pentynoyl-PADRE-J8 peptide was purified with preparative HPLC (C18 column) and detected by ESI-MS. Molecular weight: 4695, ESI-MS [M + 3H]^3+^ *m/z* 1564.8 (calc. 1566.0), [M + 4H]^4+^ *m/z* 1174.3 (calc. 1174.7), [M + 5H]^5+^ *m/z* 939.2 (calc. 940.0). [M + 5H]^6+^ *m/z* 783.1 (calc. 783.5), [M + 5H]^7+^ *m/z* 671.3 (calc. 671.7). Chromatograph C18 column 0%–100% solvent B for 50 min, t_R_ 22.9 min. Purity 97%, yield 43%. (See Supplementary Figures S1 and S2)

### 2.4. Polymer–Peptide Conjugation

4-pentynoyl PADRE-J8 (7 mg, 0.00254 mmol, 1.4 equivalent) was conjugated to the azide derivative of poly (methyl acrylate) polymer (5 mg, 0.00182 mmol, 1.0 equivalent) using copper(I)-catalyzed alkyne–azide cycloaddition (CuAAC) “click” reaction (Figure 1). Pre-activated Cu wire (60 mg, washed with concentrated H_2_SO_4_, Milli Q H_2_O, and methanol before drying under a stream of nitrogen) was used as a catalyst. After 14 h, the color of the reaction mixture changed to green and the reaction was terminated. The conjugate PMA-P-J8 was self-assembled through solvent exchange (DMF-water) and extensively dialyzed for 3 days against water. After dialysis was complete, particle diameter was measured using DLS (146 ± 8 nm) and PDI (0.190 ± 0.02). Elemental microanalysis was used to determine the N/C ratio to confirm substitution: theoretical N/C ratio: 0.206, observed N/C ratio: 0.204 (see Supplementary Table S1).

### 2.5. Immunization Study 

The immunization study was run on 14 weeks old female C57/BL6 mice. Mice were evenly divided into three groups, with five mice per group. Mice receiving the experimental vaccine candidate were orally administered with a freshly prepared dose of 30 μg of PMA-P-J8 in 30 μL of PBS on Day 1. Mice in the positive control group received the same compound plus cholera toxin B (CTB) [41]. Mice in the negative control group were administered with 30 μL PBS. Only one boost was carried out, occurring on Day 14, following the same dosing regimen. Blood samples were collected by tail bleeding on Days 0 and 14, and by heart puncture on Day 28. The samples were centrifuged for 10 min at 8000 rpm and the supernatant serum was removed and stored at −80 °C for further investigation. Saliva samples were also collected on Days 0, 14, and 28. Mice were injected intraperitoneally with 0.1% pilocarpine solution (50 μL per mouse) to induce saliva production. Saliva was then collected and stored in tubes pretreated with the protease inhibitor phenylmethylsulfonylfluoride. All samples were stored at −80 °C until further analysis.

### 2.6. Determination of Antibody Titers (IgG and IgA)

Serum and saliva samples were tested for J8-specific IgG and IgA antibodies through enzyme-linked immunosorbent assays (ELISA). First, 96 well microtiter plates were coated with carbonate coating buffer (CCB) containing 50 µg of J8 per plate, then blocked with 5% skim milk to reduce nonspecific binding. Serum and saliva samples were serially diluted in 0.5% skim milk at a 1:100 dilution for IgG and 1:4 dilution for IgA. They were then titrated to 1:2 dilution down the plate for both IgG and IgA. Secondary antibodies anti-mouse IgG and IgA (33 µL) consisting of horseradish peroxide were mixed with 100 mL of 0.5% skim milk and were then added to the plate (100 µL each well). Plates were incubated with 100 µL (each well) of OPD substrate for 20 min at room temperature. Absorbance was observed at 450 nm using a Spectra Max Microplate reader. Antibody titres were defined as the lowest dilution with an optical density of greater than the mean absorbance plus three standard deviations (SD) of control wells (pre-immunization, Day 0 sera/saliva). Statistical significance was determined by one-way ANOVA followed by Tukey’s post-hoc test. 

### 2.7. Opsonization Assay

Opsonization assays were performed with the serum samples against two different GAS clinical isolates: GC2 203 and D3840. GAS isolates were allowed to grow on freshly prepared Todd–Hewitt broth (THB) agar plates with 5% yeast extract at 37 °C for 24 h. A single colony from each isolate was transferred into freshly prepared THB (5 mL) and incubated overnight at 37 °C to obtain approximately 4.6 × 10^6^ CFU of bacteria/mL. The culture was serially diluted to 10^−2^ in PBS, from which 10 µL of culture was mixed with 5 µL of fresh, heat-inactivated serum and 70 µL of horse blood. Serum was inactivated by incubation at 50 °C for 30 min. Bacteria were then grown in the presence of serum at 37 °C for 3 h in a 96 well plate. To assess bacterial survival, 10 µL of culture material was spread on THB agar plates with 5% horse blood and incubated at 37 °C for 24 h. Colonies were then counted. The assay was performed in duplicate for each strain. Opsonic activity of the antibodies was quantified using the equation 1 − (CFU in the presence of serum/mean CFU in the presence of media)] × 100.

## 3. Results

### 3.1. Synthesis and Characterization 

**Figure 2:**
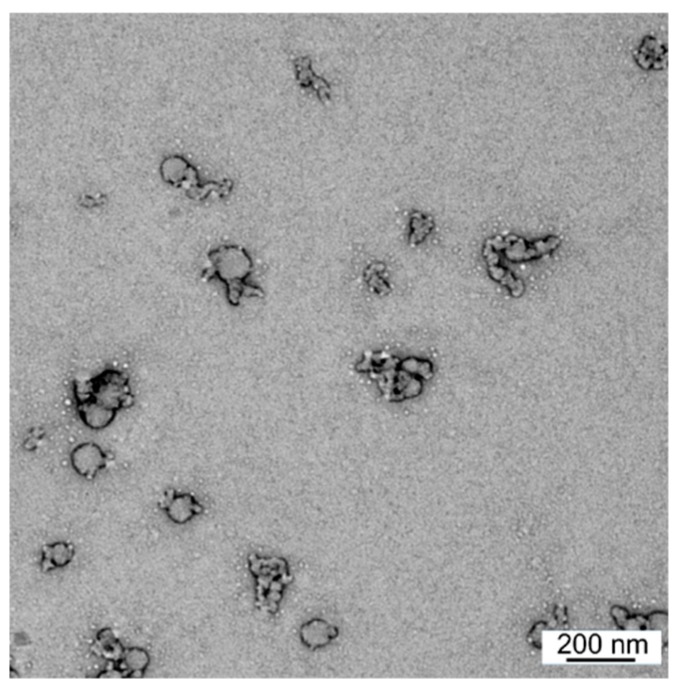
Transmission electron micrograph of PMA-P-J8 stained with 2% uranyl acetate (bar = 200 nm).

Microwave-assisted Fmoc SPPS [42] was used to synthesize the 4-pentynoyl derivative of PADRE-J8 (Figure 1). The product was conjugated to azide-substituted PMA using copper(I)-catalyzed alkyne–azide cycloaddition (CuAAC) “click” reaction [34]. The conjugate (PMA-P-J8) self-assembled via solvent exchange (DMF–water) and was extensively dialyzed for 3 days against water to remove unreacted peptide and residual copper. The substitution efficacy of the conjugation was determined through elemental analysis by comparing the C/N ratio of unsubstituted PMA with nitrogen reach conjugate (Supplementary Table S1) in the same manner as previously reported [34,40]. According to elemental analysis, quantitative substitution was achieved. DLS analysis showed that PMA-P-J8 formed monodispersed nanoparticles (146 ± 8 nm, PDI = 0.19 ± 0.02) (Supplementary Figure S3). Nanoparticle size was further confirmed through TEM (Figure 2). 

### 3.2. Immunization Study 

**Figure 3:**
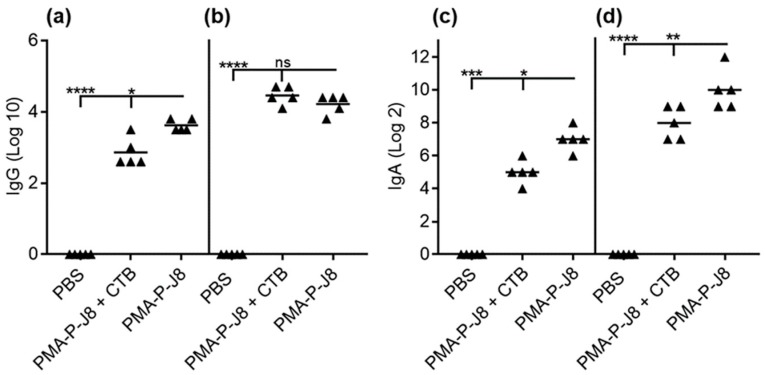
J8-specific antibody response as determined by ELISA. (**a**) J8-specific serum IgG titers after primary immunization; (**b**) J8-specific serum IgG titers after secondary immunization; (**c**) J8-specific serum IgA titers after primary immunization; (**d**) J8-specific serum IgA titers after secondary immunization. Not significant (ns) *p* > 0.05, (*) *p* < 0.05, (**) *p* < 0.01, (***) *p* < 0.001, (****) *p* < 0.0001.

**Figure 4:**
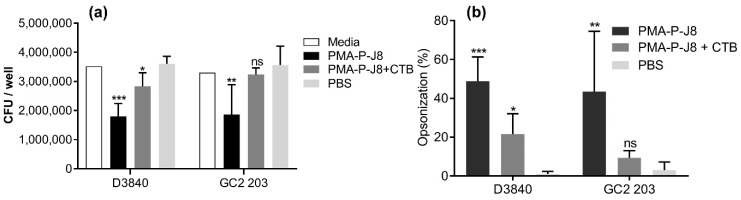
Average opsonization of D3840 and GC2 203 strains by serum collected after two immunizations with PMA-P-J8, PMA-P-J8 + CTB (cholera toxin B), or PBS expressed in (**a**) CFU and (**b**) percentages. Statistical analyses were performed using unpaired *t*-test in comparison to PBS group. Not significant (ns) *p* > 0.05, (*) *p* < 0.05, (**) *p* < 0.01, (***) *p* < 0.001.

Mice (C57/BL6 female mice, five per group) were immunized with (1) PMA-P-J8, (2) PMA-P-J8 adjuvanted with CTB (positive control), or (3) PBS (negative control). All vaccinated mice produced significantly higher antibody titers than negative control mice (PBS). Interestingly, antibody titers from blood (IgG) and saliva (IgA) collected after the first immunization showed that the polymer–peptide conjugate induced stronger immune responses on its own than when adjuvanted with CTB (Figure 3a,c). As expected, antibody titers increased in mice treated with PMA-P-J8 and PMA-P-J8 + CTB after the second immunization. Antibodies produced in mice immunized with PMA-P-J8 were opsonic (Figure 4).

## 4. Discussion

A variety of polymers have been examined as potential vaccine adjuvants or delivery systems [43,44,45]. In most cases, vaccine antigens have been formulated with polymeric nanoparticles [46], polyelectrolyte complexes [47], polymer-coated liposomes [48], or microspheres [49]. These systems, especially polyelectrolytes, have also been examined for oral vaccine delivery, as they can protect antigens from enzymatic degradation and often have mucosal adhesive and adjuvanting properties [50]. Polymers have rarely been directly conjugated to antigens to form self-adjuvanting, self-assembled nanoparticles [51,52,53,54]. Moreover, to our knowledge, such amphiphilic conjugates have never been used for the oral delivery of a vaccine. We applied our hydrophobic polyacrylate-based delivery system [33,34] to create a conjugate with the GAS B-cell epitope. We selected the J8 B-cell epitope as it has already been examined in clinical trials. The conjugate tested in humans (MJ8VAX) was composed of J8 epitope conjugated to diphtheria toxoid carrier protein as the source of T-helper epitopes. Unfortunately, the study showed that most antibody responses were generated against the carrier protein and not against J8 [28]. To avoid a similar result here, universal Pan HLA-DR-binding epitope (PADRE), which has been shown to be capable of generating efficient T-helper cell immune responses in humans [55,56], was incorporated. The conjugate (PMA-P-J8) was produced using standard SPPS and “click” reaction, and quantitative substitution efficacy was achieved. PMA-P-J8 self-assembled into monodispersed nanoparticles (~150 nm), in contrast to previously reported polyacrylate conjugates, which have been rather polydispersed or formed microparticles [34,37,57]. The monodispersity and lack of aggregation of PMA-P-J8 might have been related to the excellent substitution efficacy, in addition to the influence of hydrophilic PADRE-J8 on the amphiphilic properties of the conjugate. 

Mice treated with PMA-P-J8 produced high levels of both systemic IgG and mucosal IgA following a single immunization (Figure 3). Surprisingly, significantly lower systemic and mucosal antibody titers resulted from mice immunized with PMA-P-J8 adjuvanted with CTB, the standard control adjuvant used for oral immunizations. The unexpected lower vaccine efficacy may have been related to CTB’s ability to trigger tolerance upon oral administration [58,59,60]. However, when mice were boosted with a second dose of vaccine, both PMA-P-J8 and PMA-P-J8 + CTB groups generated higher systemic and mucosal antibody titers in comparison to single immunization, suggesting that oral tolerance had not occurred. Importantly, systemic antibodies (IgG) generated against PMA-P-J8 showed potent opsonic activity against GAS clinical isolates (Figure 4). In contrast, despite inducing similar IgG titers following boost immunization, PMA-P-J8 + CTB was not able to trigger the production of highly opsonic antibodies. Notably, PMA-P-J8 induced strong oral immune responses against a weak peptide antigen after a single dose (30 µg), while other oral delivery systems for peptide antigens have required multiple doses, and hundreds of micrograms in total [61]. 

## 5. Conclusions

All currently approved oral vaccines utilize attenuated whole pathogens, as no effective oral delivery system for subunit-based vaccines exists. Similarly, there is a lack of potent oral adjuvants. Here, we demonstrated that a polymer-based delivery system can induce strong systemic and mucosal immune responses after a single low-dose immunization without the help of an external adjuvant. While the produced antibodies were opsonic following a second immunization, further GAS challenge experiments will be required to confirm vaccine efficacy. This strategy opens the door for the oral delivery of subunit vaccines against a variety of infectious diseases. 

## Supplementary Materials

The following are available online at https://www.mdpi.com/2076-393X/8/1/23/s1, Table S1: Element microanalysis for PMA and PMA-P-J8, Figure S1: ESI-MS Spectrum of 4-petynoyl derivative of PADRE-J8 Peptide, Figure S2: Analytical RP-HPLC chromatogram image of PADRE-J8 with alkyne moiety, Rt = 22.9 min, Figure S3: DLS spectra of particle PMA-P-J8 size distributions by intensity.
