# Supplementary material for: Polyacrylate–Peptide Antigen Conjugate as a Single-Dose Oral Vaccine against Group A Streptococcus
Source: Vaccines (Basel). 2020 Jan 13;8(1):23. doi: 10.3390/vaccines8010023 (PMC7157655; doi:10.3390/vaccines8010023)
Supplement: Supplementary file 1 [file vaccines-08-00023-s001.pdf]

vaccines

MDPI

Supporting Information

Polyacrylate-peptide antigen conjugate as a single-dose oral vaccine against Group A Streptococcus

Mohammad Omer Faruck[1], Lili Zhao[1], Waleed M. Hussein[1,2], Zeinab G. Khalil[3], Robert J. Capon[3], Mariusz Skwarczynski[1,*], Istvan Toth[1,3,4*]

1 School of Chemistry and Molecular Biosciences, The University of Queensland, St Lucia, Brisbane, QLD 4072, Australia;
2 Pharmaceutical Organic Chemistry Department, Faculty of Pharmacy, Helwan University, Helwan, Egypt;
3 Institute for Molecular Bioscience, The University of Queensland, St. Lucia, QLD 4072, Australia;
4 School of Pharmacy, The University of Queensland, Woolloongabba, Brisbane, QLD 4102, Australia;
* Correspondence: e-mail: m.skwarczynski@uq.edu.au, i.toth@uq.edu.au

Table 1. Element microanalysis for PMA and PMA-P-J8.

		N %	C %	N/C ratio
PMA	Theoretical Value	1.53	55.00	0.028
		10.27	49.81	0.206
PMA-P-J8	Experimental Value	10.19	49.80	0.204

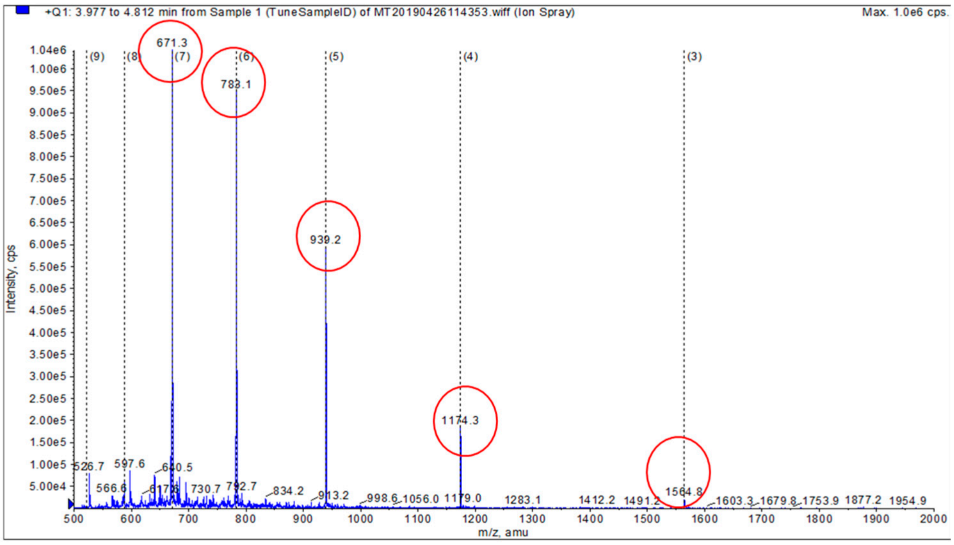

Figure 1. ESI-MS Spectrum of 4-petynoyl derivative of PADRE-J8 Peptide.

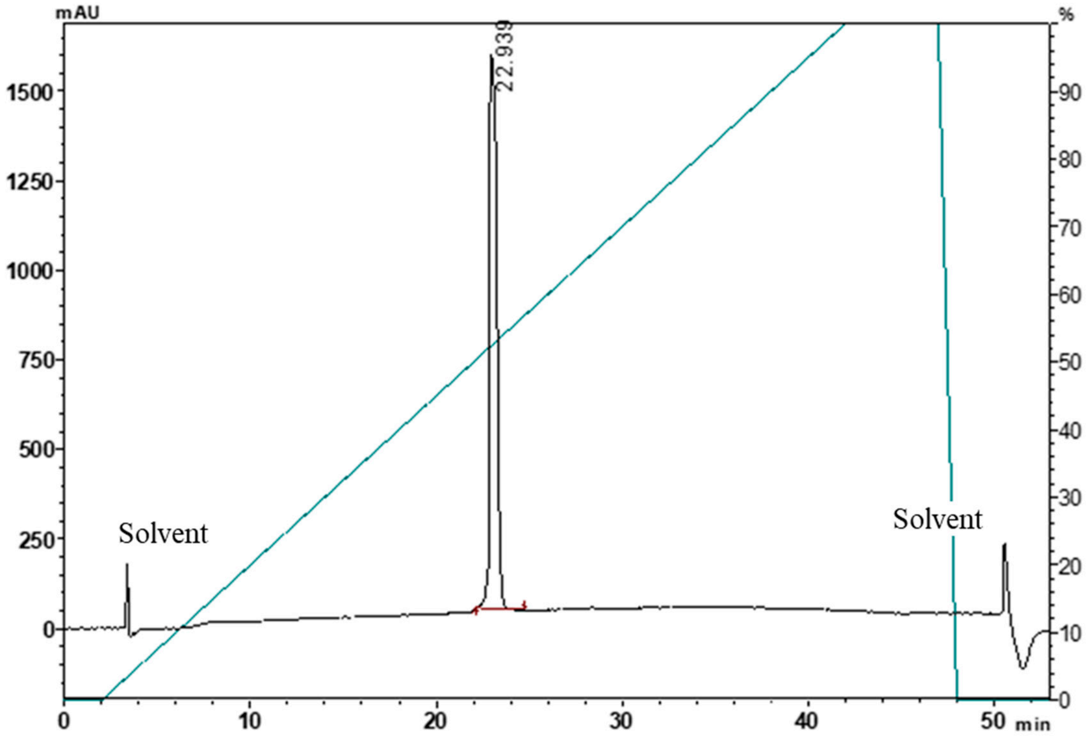

Figure 2. Analytical RP-HPLC chromatogram image of PADRE-J8 with alkyne moiety, Rt=22.9 min.

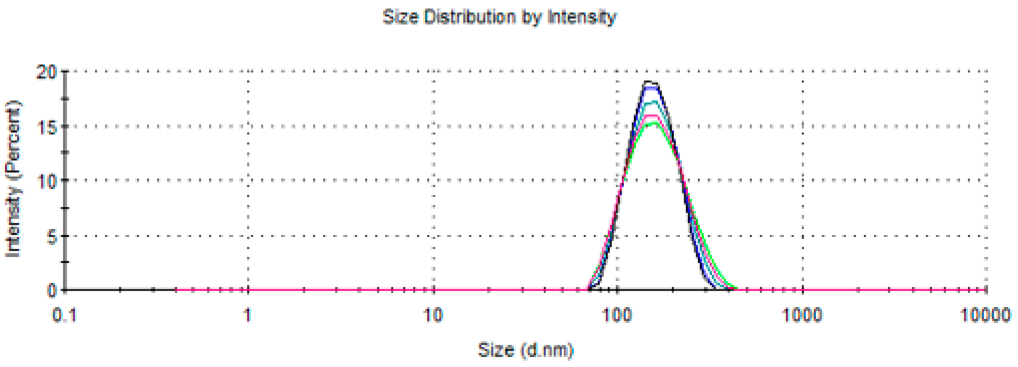

Figure 3. DLS spectra of particle PMA-P-J8 size distributions by intensity.
